# Date Palm Fiber Agricultural Residue Valorization in Rotationally Molded LLDPE Single- and Multilayered Composites for Engineering Applications

**DOI:** 10.1021/acsomega.6c01931

**Published:** 2026-08-20

**Authors:** Abu Saifullah, Ataur Rahman, Salim Reza, Jibu Joseph, Lei Wang, Andrew R. Pickford, Hom N. Dhakal

**Affiliations:** † Portsmouth Centre for Advanced Materials and Manufacturing (PCAMM), School of Electrical and Mechanical Engineering (SEME), 6697 University of Portsmouth, Portsmouth PO1 3DJ, U.K.; ‡ Matrix Polymers, Northampton NN3 6AX, U.K.; § Centre for Enzyme Innovation, Preventing Plastic Pollution with Engineering Biology (PE3B) Mission Hub, School of the Environment and Life Sciences, University of Portsmouth, Portsmouth PO1 2DT, U.K.

## Abstract

Waste material valorization in rotational molding is gaining attention for modifying mechanical properties and reducing the use of virgin polymers in plastic components. This study investigated, first of its kind, the use of date palm fibers (DPF), an agricultural residue available abundantly in the Middle East and North Africa region, in linear low-density polyethylene (LLDPE) composites via rotational molding. Roto-molded DPF/LLDPE composites were manufactured with different fiber contents of 5–20 wt % in single-, double-, and triple-layered structures combining only LLDPE and fiber-incorporated LLDPE layers. Double- and triple-layered molding cycles were 10–27% longer than single-layer ones. These multilayered composites showed higher densities compared to single layers, although fiber addition overall reduced composite density. Rheological data and SEM images confirmed good fiber dispersion in the polymer matrix. Differential scanning calorimetry (DSC) and X-ray diffraction (XRD) analyses confirmed no changes in crystal structure. DPF addition slowed crystallization, reduced crystallinity and thermal stability compared to only LLDPE samples. The tensile modulus increased by 34–49% in single- and double-layered composites with higher fiber content. However, triple-layered structures demonstrated balanced mechanical performance, retaining a tensile modulus closer to only LLDPE while showing the highest impact strength compared to all composites and even surpassing LLDPE. DPF increased hydrophilicity and thermal insulation in single-layered composites. Double- and triple-layered structures are expected to reduce moisture uptake while further enhancing thermal insulation. These results support the effective valorization of DPF in roto-molded composites, enabling sustainable solutions tailored to specific product performance requirements.

## Graphical abstract

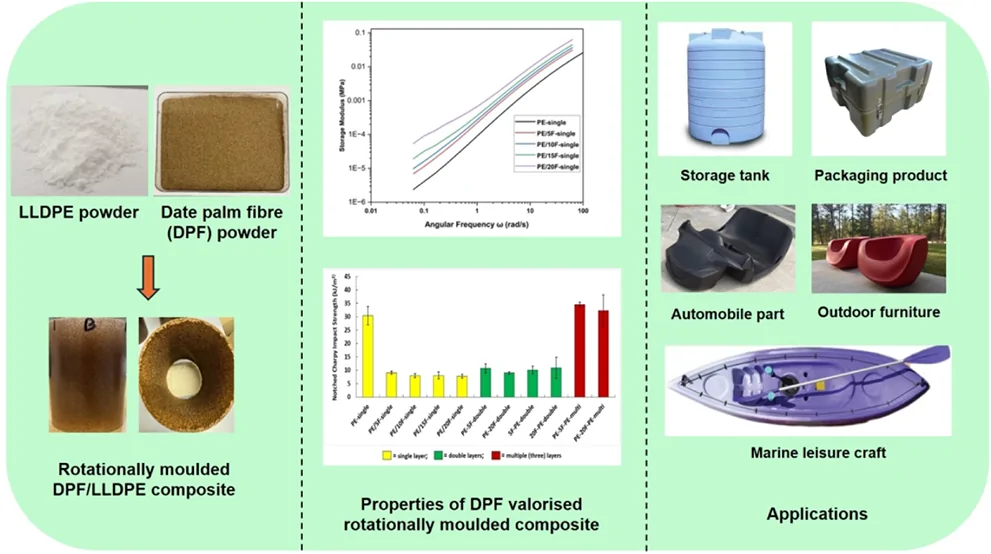

## 1 Introduction

Plastics have had a transformative impact on modern life due to their unique properties and versatility across a wide range of applications. Because of the ubiquitous use of plastics, global annual plastic production has increased sharply, exceeding 450 Mt/year. This represents an increase of nearly 300-fold compared with the plastic production level in the 1950s.^1^ Depending on the size, shape, and production volume of plastic products, a variety of plastic processing techniques exist, such as extrusion, injection molding, thermoforming, compression molding, blow molding, and rotational molding, among others.

Rotational molding (roto-molding) is a unique polymer processing technique suitable for producing large, hollow, seamless, and complex structures with single- or multilayer designs.^2–4^ The industry is growing at 6% annually and is expected to reach €8.5 billion by 2031.^5^ Compared to compression, injection, or blow molding, it offers several advantages, including low-cost tooling, simple operation, minimal waste, and stress-free parts due to its pressure-free process.^6–8^ Key features such as zero shear stress, long cycle times, and slow cooling influence the final microstructure and performance of the plastic products produced. Single- or multilayer products can be made without seams or joints within the same processing cycle. The process involves four steps: loading, heating, cooling, and demolding.^8^ Common products include large storage tanks, furniture, toys, automotive parts, boats, and insulation panels. Polyethylene (PE) is the most widely used for its good melt flow and heat resistance; PP, PA, PC, and polyesters are also used. As most polymers are thermoplastic and recyclable, the process supports sustainability. This industry is currently focusing on recycling, using postconsumer polymers, and developing biopolymers or biocomposites from natural fibers or agricultural residues, aligning with the UN SDGs 7, 8, 9, 12, and 13.^9^

The use of natural fibers in sustainable roto-molded products is being increasingly investigated due to their renewability, low cost, biodegradability, and insulating properties. Incorporating fibers can improve mechanical behavior and reduce virgin polymer usage. Studies mainly focus on polyethylene (PE) systems (LLDPE, LDPE, HDPE).^9^ PE is a semicrystalline,^10^ ductile material and can be easily processed in the rotational molding process. Fiber size strongly affects polymer mixing and performance; powders (355–500 μm) enhance mechanical properties^11,12^ through better dispersion, while long or short fibers often agglomerate due to the low-shear, pressure-free nature of rotational molding. Fiber content is typically limited to 30–40%, as higher contents reduce uniformity and strength. Fiber–polymer blending, conducted before molding, is done by either dry or melt blending. In dry blending, fiber and polymers are mixed manually and/or with a shear mixer without heating, while melt blending involves mixing fibers into molten polymer under heat and pressure. For roto-molding, dry blending is simpler, faster, and often yields better thermal and mechanical properties, since melt blending can degrade fibers.^12^ The influences of natural fibers in rotationally molded polymer composites and their mechanical and other properties are described in Section 1.1, Figure 1.

**Figure 1:**
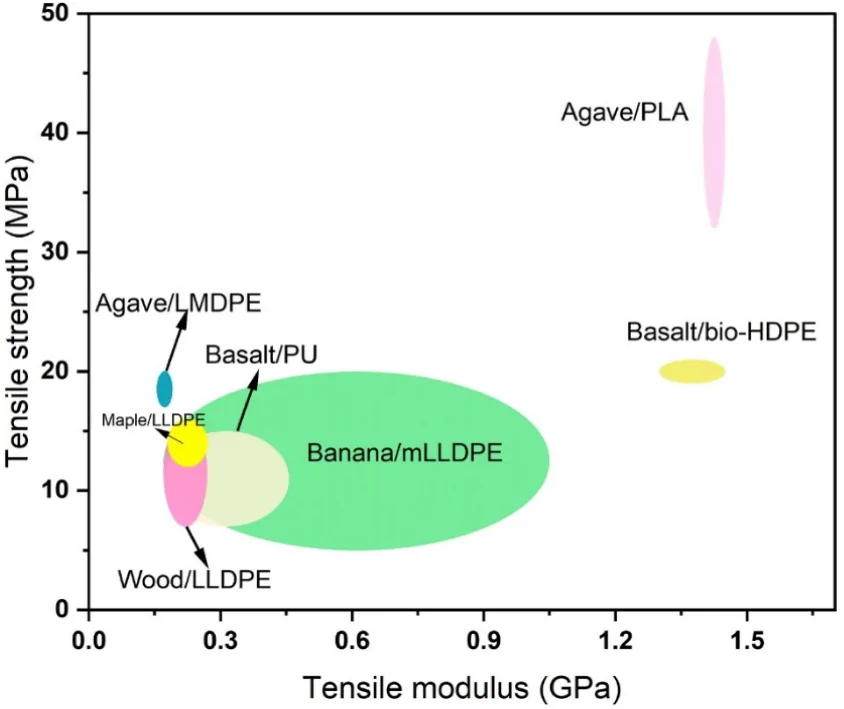
Tensile properties of various natural fiber polymer or agricultural biomass/polymer composites manufactured by the rotational molding process. Data used to create this figure were collected from the literature, and the respective references are cited in Section 1.1.

Beyond the use of virgin natural fibers, there is a growing interest in valorizing agricultural residues and agro-industrial byproducts in rotational molding.^9^ Agricultural residues, generated during harvesting (e.g., rice and wheat straw) or crop processing (e.g., fruit peels, sugarcane bagasse, date palm fibers), are often underused or improperly disposed of, causing environmental issues.^13,14^ These residues can instead serve as reinforcements or fillers in polymer matrices,^15,16^ promoting resource efficiency, the circular bioeconomy, and sustainable development. Among them, date palm fibers (DPFs), byproducts of pruning and fruit harvesting, offer promising potential for use as fillers or reinforcements in roto-molded plastics. To date, no research has explored their use in this application. DPFs are abundant (over 100 billion trees globally), mainly in the Middle East and North Africa (MENA), with southern Algeria producing 26–40 kg of waste per tree annually (≈4 Mt globally, including ≈200,000 tonnes in Saudi Arabia and 2.6–2.8 Mt in MENA).^13,15,17^ They are 900–5800% cheaper than coir, hemp, bamboo, and sisal fibers,^18,19^ lightweight (density ≈ 0.93 g/cm^3^), and contain ∼40% cellulose with balanced hemicellulose and lignin contents (25–30% each). This composition provides reasonable tensile strength (58–309 MPa) and tensile modulus (2–7.5 GPa), high elongation (5–19%), and good hydrophobicity due to a higher lignin content.^20,21^ In the literature, many studies have reported the incorporation of DPFs into epoxy;^17^ unsaturated polyester-based hybrid fiber-reinforced composites;^22^ polyvinyl chloride/LDPE;^23^ polyvinyl chloride/HDPE;^24^ and polyvinyl chloride/LDPE/acrylonitrile butadiene rubber copolymer ternary polymer composites^25^ and their properties have been investigated in detail.

Considering the advantageous properties and availability of DPFs, along with the growing need to utilize agricultural residues in roto-molded biocomposites to reduce virgin polymer use, this study investigates the valorization of DPFs in rotational molding for the first time. DPFs were dry-blended with LLDPE at various fiber contents (5, 10, 15, and 20 wt %) to produce single-layer DPF/LLDPE composites. Additionally, double- and multilayered composites were fabricated using different fiber/polymer weight ratios and layer sequences. The objectives were to manufacture single-, double-, and multilayered DPF/LLDPE composites and evaluate their rheological, density, mechanical, thermal, morphological, wettability, and thermal conductivity characteristics. During the roto-molding process, the outer layer of a plastic product is in contact with the mold; therefore, a smooth outer layer is formed. For the fiber-incorporated single-layer plastic structure, the outer surface is smooth, while the inner surface is fiber-rich, rough, and uneven. For applications such as chemical or water storage, the rough inner surface and its hydrophilicity can be problematic. A double-layered configuration in which a smooth, hydrophobic polymer layer is added inside addresses this issue. In contrast, for applications where outer appearance matters (e.g., outdoor furniture, boat hulls), a polymer-only outer layer can be formed first, followed by a fiber-incorporated inner layer. A multilayered (triple) structure can sandwich the fiber/polymer layer between two polymer-only layers, offering both aesthetic appeal and improved functional performance. To explore the unique benefits of double- and triple-layered DPF/LLDPE roto-molded composites, this work also measured and identified modifications in the mechanical, thermal, and morphological properties of layered roto-molded structures relative to single-layered DPF/LLDPE composites.

### 1.1 Influence of of Natural Fibers and Agricultural Waste Biomass on Rotationally Molded Composites

Figure 1 depicts the range of tensile properties of fiber-incorporated rotationally molded composites. Incorporating natural fibers into roto-molded plastics generally enhances tensile and flexural strengths. Maple (20 wt %),^11^ flax (5 wt %),^26,27^ hemp (5 wt %),^27^ and bamboo (up to 15 wt %)^28,29^ fibers in LLDPE showed improved mechanical performance. Similarly, rice husk in HDPE,^30^ agave (up to 15 wt %) in LMDPE,^31^ and basalt fiber powder in PU enhanced tensile strength. Conversely, coir in bio-LLDPE^32^ and wheat bran in LLDPE^33^ reduced tensile properties. Improved impact strength was reported for wood fiber in LDPE^26,34^ and coir in bio-LLDPE.^32^ Mechanical performance is further enhanced by chemical treatments of the fiber or matrix that improve interfacial adhesion. NaOH treatment removes lignin and hemicellulose, increasing fiber roughness and bonding, as seen in coir- and bamboo-LLDPE composites.^35^ Maleic anhydride-grafted polyethylene (MAPE) acts as a coupling agent, markedly improving strength and water resistance;^11,32^ MAPE treatment of agave and coir fibers increased flexural strength, modulus, and impact strength by 100%, 34%, and 46%, respectively.^32^ Other treatments, including silane, benzoylation, and peroxide treatment, have also been reported.^36^

Banana fiber, collected from banana peels and different parts of banana plants, has been studied in the roto-molding process. Multilayered roto-molded plastics were manufactured with banana fiber using both dry^37^ and melt^2^ blending processes. In the melt-blended multilayered banana (20 wt %)/mLDPE samples, the multilayered structures showed significantly improved mechanical properties compared to single-layered structures, although they were lower than those of mLDPE alone, especially in terms of impact performance. Ramkumar et al.^38^ found 10 wt % fiber content optimal in dry-blended banana/LLDPE composites based on FTIR, DSC, and water absorption results. In the currently available literature, a limited amount of work has been performed to understand property and process relationships of roto-molded agricultural residue-incorporated composites. Further research is needed to evaluate other agricultural residues (for example, date palm fiber) for roto-molded biocomposites and their engineering applications.

## 2 Materials and Methods

### 2.1 Materials

Rotational molding-grade LLDPE polymer powder with an MFI (melt flow index) of 10 (2.16 kg, 190 °C) and a density of 0.935 g/cm^3^ was collected from Matrix Polymer Ltd., UK. The LLDPE polymer powder size was distributed between ∼300 and 500 μm. Long date palm fibers (leaf sheath) were sourced from Al-Ahsa, Eastern Province, Saudi Arabia, with a density of 0.9–1.2 g/cm^3^, a breaking tensile strength of 170–275 MPa, a tensile modulus of 5–12 GPa, and an elongation of 5–10%.^15,39,40^ DPFs were cleaned, dried, chopped into smaller sizes, and subsequently cryo-ground with a benchtop centrifugal mill (Retsch Centrifugal Mill ZM 300) into DPF fiber powders with a particle size range of 350–500 μm (Figure 2).

**Figure 2:**
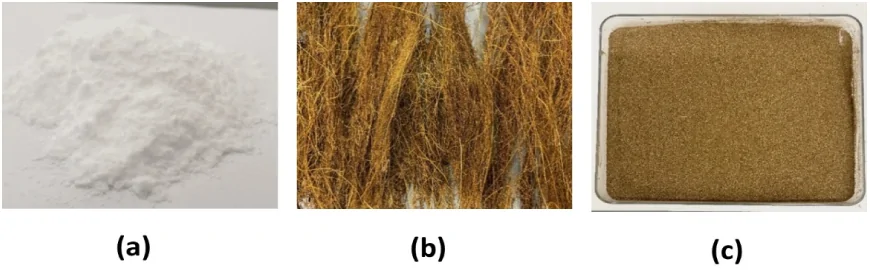
Images of the polymer and date palm fiber: (a) LLDPE polymer powder, (b) long date palm fibers (DPF), and (c) DPF powder.

### 2.2 Rotational Molding of Date Palm Fiber/LLDPE Composites

Date palm fiber (DPF) powders were dried in an oven at 90 °C for 2 h before dry-blending them with LLDPE powder. In the dry-blending process, a two-step mixing process was applied: an initial manual/hand mixing of the required amount of fiber and polymer powders, followed by a shear mixing process, so that good mixing of the fiber and polymer powders could be achieved. For manufacturing of the composites, a benchtop, lab-scale uniaxial roto-molding equipment (RotoRocket, Figure 3) was used, with a steel cylindrical mold of 145 mm in length, 90 mm in diameter, and 2 mm thickness, rotating at a speed of 12 rpm and equipped with two integrated electric heaters. The mold rotation direction was oriented toward the heaters of the uniaxial roto-molding machine. Single, double, and multilayered (triple) date palm fiber-filled roto-molded LLDPE composites were manufactured as follows. Antioxidants were used at a 0.2 wt % ratio for all types of manufactured samples. Internal air temperatures (IAT) were monitored with the RotoRocket K-kord datalogger during the manufacturing process of all types of DPF-filled LLDPE composites. The outer surface of the produced DPF/LLDPE composites was in contact with the mold, while the inner surface was the inner layer of the plastics and was not in contact with the mold during the manufacturing process.

**Figure 3:**
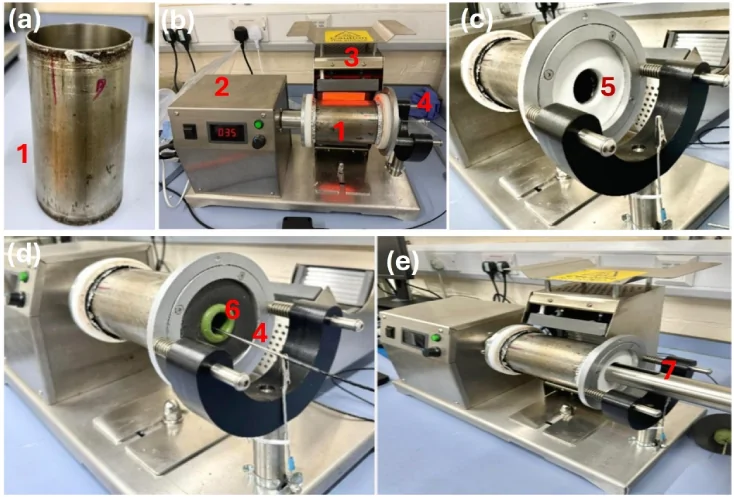
Uniaxial rotational molding equipment details: (a) steel cylindrical mold (1); (b) mold (1) loaded in the machine with rotation control (2), heating on (3), and mold internal air temperature (IAT) data logger inserted (4); (c) side view of the machine showing the vent pipe hole (5); (d) the machine with the vent pipe inserted through the vent pipe hole (6) and the IAT data logger (4) inserted through the vent pipe inside the mold; (e) the drop-box (7) inserted through the vent pipe hole to add additional layer (2nd and 3rd) materials without removing the mold from the molding process.

#### 2.2.1 Single-Layered Composites

Different DPF powder ratios (5, 10, 15, and 20 wt %) were added to the LLDPE polymer powder to manufacture single-layered composites with a thickness of 3 mm. The required amount of polymer/fiber powder mix was added to the cylindrical steel mold, heated up to a peak internal air temperature (PIAT) of 190 °C, followed by a fan-assisted cooling cycle down to 50 °C IAT and finally, the samples were demolded (Figures 3 and 4a, Table 1).

**Table 1:** Rotationally Molded DPF/LLDPE Composite Manufacturing Details and Codes^a^

Composites and Codes		Structure Configurations	Roto-molding Parameters
**Single Layered**	PE-single	PE/Fiber mixture at 5, 20, 25, and 20 wt % fiber ratios (w/w); 3 mm thick.	Heating – PIAT of 190 °C; cooling to 50 °C.
	PE/5F-single		
	PE/10F-single		
	PE/15F-single		
	PE/20F-single		
**Double Layered**	PE-5F-double	PE only outer layer (1 mm); PE/fiber mixture inner layer (2 mm); only 5 and 20 wt % fiber ratios.	Heating (IAT) – outer layer 140 °C; inner layer 190 °C; cooling to 50 °C.
	PE-20F-double		
	5F-PE-double	PE/fiber mixture outer layer (2 mm); PE only inner layer (1 mm); only 5 and 20 wt % fiber ratios.	
	20F-PE-double		
**Multilayered (triple)**	PE-5F-PE-multi	PE only outer layer (1 mm); PE/fiber mixture middle layer (1 mm); PE only inner layer (1 mm); only 5 and 20 wt % fiber ratios.	Heating (IAT) – outer layer 130 °C; middle layer 140 °C; inner layer 190 °C; cooling to 50 °C.
	PE-20F-PE-multi		

a IAT: internal air temperature; PIAT: peak internal air temperature.

**Figure 4:**
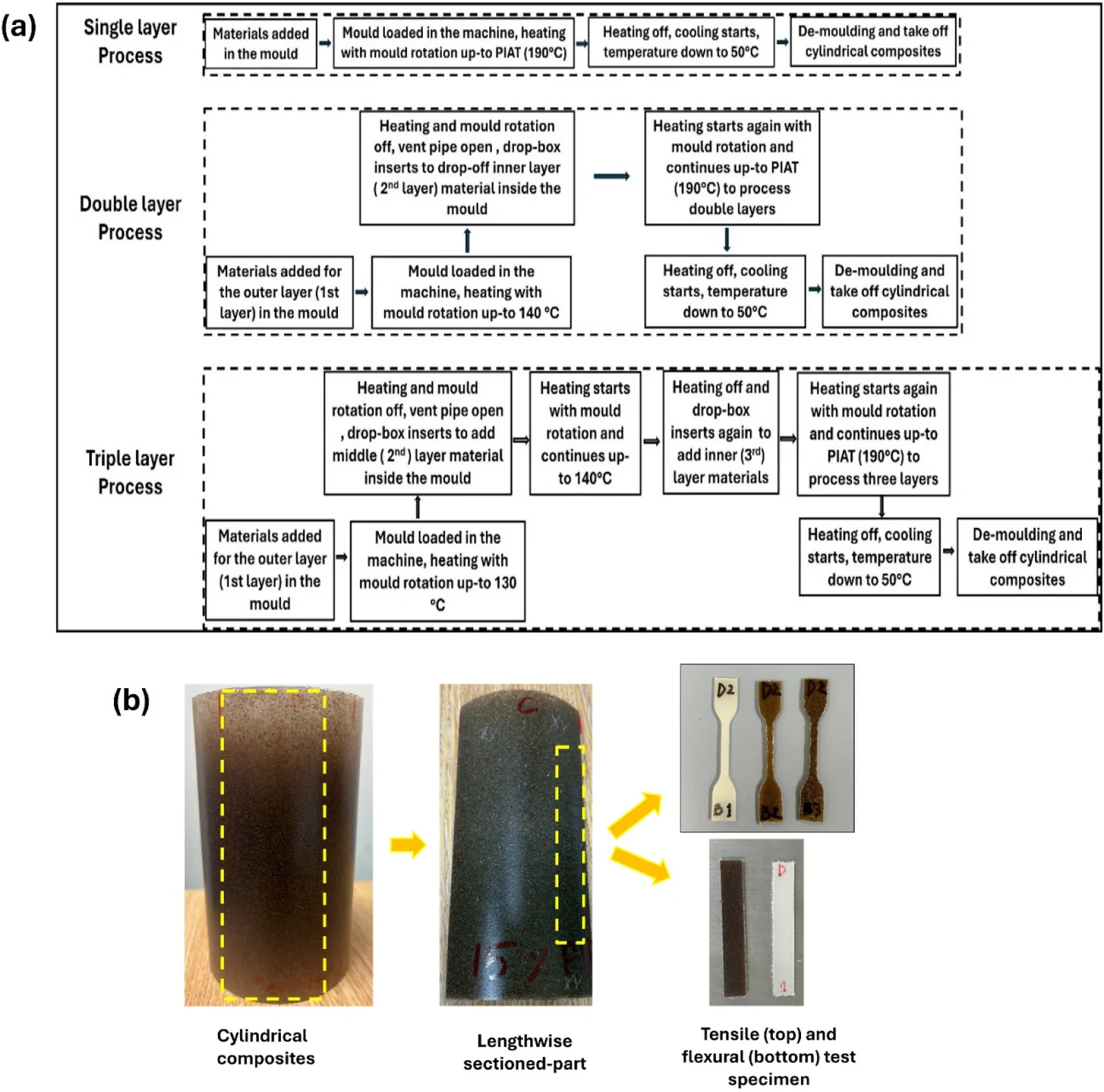
(a) Single-, double-, and triple-layered rotational molding processes for DPF/LLDPE composites; (b) test specimen preparation procedure from cylindrical-shaped biocomposites.

#### 2.2.2 Double-Layered Composites

Only 5 and 20 wt % fibers were used in producing double-layered composites with a 3-mm thickness, wherein a PE only layer (1 mm thick) was created either inside or outside, with a 2-mm-thick fiber/polymer mixed layer outside or inside, respectively. According to the different configurations, the first layer (outer layer) was heated up to 140 °C, then the heater was turned off to add the second layer (inner layer) material using a manual drop box in the equipment, with a subsequent second heating up to 190 °C, followed by a cooling process before demolding the double-layered samples (Figures 3 and 4a, Table 1).

#### 2.2.3 Multilayer (Triple) Composites

In triple-layered structures, the fiber/polymer mixed layer (only 5 and 20 wt % fibers; thickness 1 mm) was sandwiched between outside and inside PE-only layers (1 mm thickness each). The first layer (outer layer) material was added and heated to 130 °C. After that, the second (middle layer) and third (inner layer) materials were added and heated one after another to 140 and 190 °C, respectively. During the addition of the second and third layer materials using the manual steel drop-box, the heater was kept off to add the materials smoothly (Figures 3 and 4a, Table 1). Details of the DPF/LLDPE composite samples and codes are given in Table 1.

### 2.3 Characterizations

#### 2.3.1 Rotational Molding Cycle Time Analysis

The overall rotational molding cycle time and its changes during the manufacturing of single/double/multilayered composites were analyzed using the roto-molding K-Kord process control thermocouple data.

#### 2.3.2 Rheological Tests

The TA HR3 hybrid rheometer (TA Instruments, USA) was used to conduct rheological analysis at 190 °C. A 25 mm stainless-steel parallel plate with a 1 mm geometry gap test arrangement was utilized in the frequency sweep tests, with angular frequency ranging from 0.1 to 100 rad/s at a 1% strain rate.

#### 2.3.3 Density Measurements

The water-immersion method was used to measure the density of each roto-molded composite in accordance with ISO 1183-1:2019. At least five repetitions were conducted to measure the average density of each biocomposite.

#### 2.3.4 Mechanical Properties Analysis—Tensile, Flexural, and Impact Properties

Tensile properties of DPF/LLDPE composites were tested according to the ISO 527-2 test standard,^41^ using a 50 mm/min test speed. The produced cylindrical composites were sectioned lengthwise into four parts with a bandsaw (Figure 4b). Tensile test specimens were subsequently punched in the longitudinal direction according to the ISO 527-2 Type 5A specimen dimensions with the help of a puncher. Flexural tests were carried out in a three-point bending arrangement according to the ISO 178 test standard.^42^ The flexural test specimens were cut from sectioned parts using a bandsaw, with a length of 80 mm, a width of 10 mm, and a thickness of 3 mm. The test speed and span length were 5 mm/min and 50 mm, respectively. The adopted tensile and flexural test speeds were consistent with those reported in previous studies.^10,43–45^ A 10 kN load cell-attached ZwickRoell Z010 (Ulm, Germany) universal test equipment was utilized to conduct both tensile and flexural tests, with at least five repetitions for each composite type. Notched Charpy impact test specimens, 80 mm long, 10 mm wide, and 3 mm thick, were first cut with the help of a bandsaw from sectioned parts, and then a notch was created with the help of a notch-inserting element. They were tested in a manual Charpy impact tester according to the ISO 179 test guidelines.^46^ For calculating the Charpy impact strength, eq 1 was used. Five specimens were tested, and the average absorbed impact energy to break the tested specimens was calculated and reported. Figure 4b is provided to show the test specimen preparation procedure. All tensile, flexural, and impact specimens were flat because the maximum width of the specimens was 10 mm, and the effect of the cylindrical shape was negligible for each specimen. Therefore, there was no problem gripping the samples for tensile, flexural or impact testing. In addition, all tested specimens were created and tested following the same procedures so that the results can be compared.

1 $$a_{cN}=\frac{W_{c}}{h\times b_{N}}$$

where *a_cN_* is calculated notched Charpy impact strength; *W_c_* is the energy absorbed during the breaking of test specimen; *h* and *b_N_* are thickness and remaining width after notch of tested specimens, respectively.

#### 2.3.5 Scanning Electron Microscopy (SEM) Images

SEM images of the cross sections of single-, double-, and triple-layer DPF/LLDPE composites were taken to investigate the morphology and fiber distribution in the PE polymer matrix. Specimens broken in Charpy-impact tests were also investigated to understand the fracture mechanisms of the composites under impact loads. The specimens were gold-coated before taking SEM images with a ZEISS SEM (EVO 10 MA, Carl Zeiss AG, Germany).

#### 2.3.6 Differential Scanning Calorimetry (DSC)

For the DSC analysis of the produced DPF/PE composites, a heat-cool-heat method was applied at a 10 °C/min heating or cooling rate, wherein the first and second heating cycles were performed from 20 to 180 °C, while the cooling cycle was conducted from 180 to 20 °C. The TA Q100 DSC (TA Instruments, USA) was used to conduct the DSC analysis. From the DSC tests, crystallinity (%) values were calculated using eq 2,

2 $$\mathrm{Crystallinity},X_{C}\;(\%)=\frac{\Delta H_{f}}{\Delta H_{f0}}\times 100\%$$

where, Δ*H*_*f*0_ is termed for 100% LLDPE crystalline sample, found its value as 293 J/g^2^ in the literature, whereas Δ*H_f_* provides heat of fusion values of each gram of composite samples measured from DSC tests.

#### 2.3.7 Thermogravimetric Analysis (TGA)

Thermal degradation behavior was analyzed for the developed composites using a TA Q50 TGA (TA Instruments, USA) under a N_2_ gas atmosphere from 30 to 700 °C at a heating rate of 20 °C/min.

#### 2.3.8 XRD Analysis

To analyze the crystal structure of LLDPE and any changes that occurred due to the addition of date palm fibers to the PE polymer matrix, single-layered DPF/LLDPE samples were XRD-tested using a Malvern Panalytical Empyrean 3 X-ray diffractometer (Malvern, Worcestershire, UK) to capture the crystal structure from 5° to 40° 2θ angles, employing Cu Kα X-ray emission with a wavelength of 0.15406 nm, at 45 kV and 40 mA.

#### 2.3.9 Water Contact Angle Measurements

To investigate the surface wettability of the composite samples, a KSV CAM-101 (KSV Instruments, Finland) water contact measurement equipment was utilized to release water droplets at different locations on the tested composite samples and subsequently measure the contact angles between the water droplets and the surfaces. An average water contact angle was reported based on 10 different measurements for single-layered composite samples only.

#### 2.3.10 Thermal Conductivity

Thermal conductivity was measured only for single-layered composites. The hot-wire method, according to the ISO 8894-1 test standard, was used to measure the conductivity with the test equipment (QTM-500, PD-11, Kyoto Electronics, Japan). For calculating thermal insulation, the tested composite sample thicknesses were divided by the measured thermal conductivity values of the respective composites.

## 3 Results and Discussion

### 3.1 Rotational Molding Cycle Time Analysis of DPF/LLDPE Composites

Table 2 presents rotational molding cycle time details for DPF-filled LLDPE composites, derived from the internal air temperature profiles of the moldings, shown in Figure 5. The cycle time data confirmed that both DPF incorporation and layered configurations have effects on the total molding cycle durations. In single-layered structures, increasing DPF content reduced the overall molding cycle time. The effect was primarily due to a shorter cooling phase in the molding cycle, while the heating cycles remained unchanged irrespective of DPF content in single-layered DPF/LLDPE composites. The shortened cooling time was mainly associated with the use of a lower overall quantity of polymer for high-fiber-content DPF-added roto-molded composites, which release heat and solidify quickly compared to using a larger quantity of polymer. This effect has been reported previously in the literature from previous works.^2^ Depending on the number of layers in the layered structures, the overall molding cycle time increased by 10–16% for double-layered and 23–27% for triple-layered structures. The cycle time increase was related to the time taken for the addition of the second and third layers in the roto-molded DPF/LLDPE composites. For both double- and triple-layered structures, the quicker cooling process and the overall lower molding duration were also seen with higher fiber content in the DPF/LLDPE composites.

**Table 2:** Roto-Molding Cycle Time Details of Produced DPF/LLDPE Composites

Composites	Heating Cycle Time (mm:ss)	Cooling Cycle Time (mm:ss)	Whole Cycle Time (mm:ss)	Cycle Time Changes (%) Compared to PE-Single)
PE-single	10:47	13:08	23:55	-------
PE/5F-single	11:11	12:95	24:06	(+) 2.16%
PE/10F-single	11:30	12:77	24:07	(+) 2.20%
PE/15F-single	11:19	12:07	23:26	(−) 1.23%
PE/20F-single	11:31	11:71	23:02	(−) 2.25%
PE-5F-double	14:20	11:88	26:08	(+) 10.74%
PE-20F-double	14:23	11:79	26:02	(+) 10.48%
5F-PE-double	15:07	12:32	27:39	(+) 16.30%
20F-PE-double	15:07	11:44	26:51	(+) 12.56%
PE-5F-PE-multi	17:00	13:02	30:02	(+) 27.47%
PE-20F-PE-multi	17:03	12:03	29:06	(+) 23.40%

**Figure 5:**
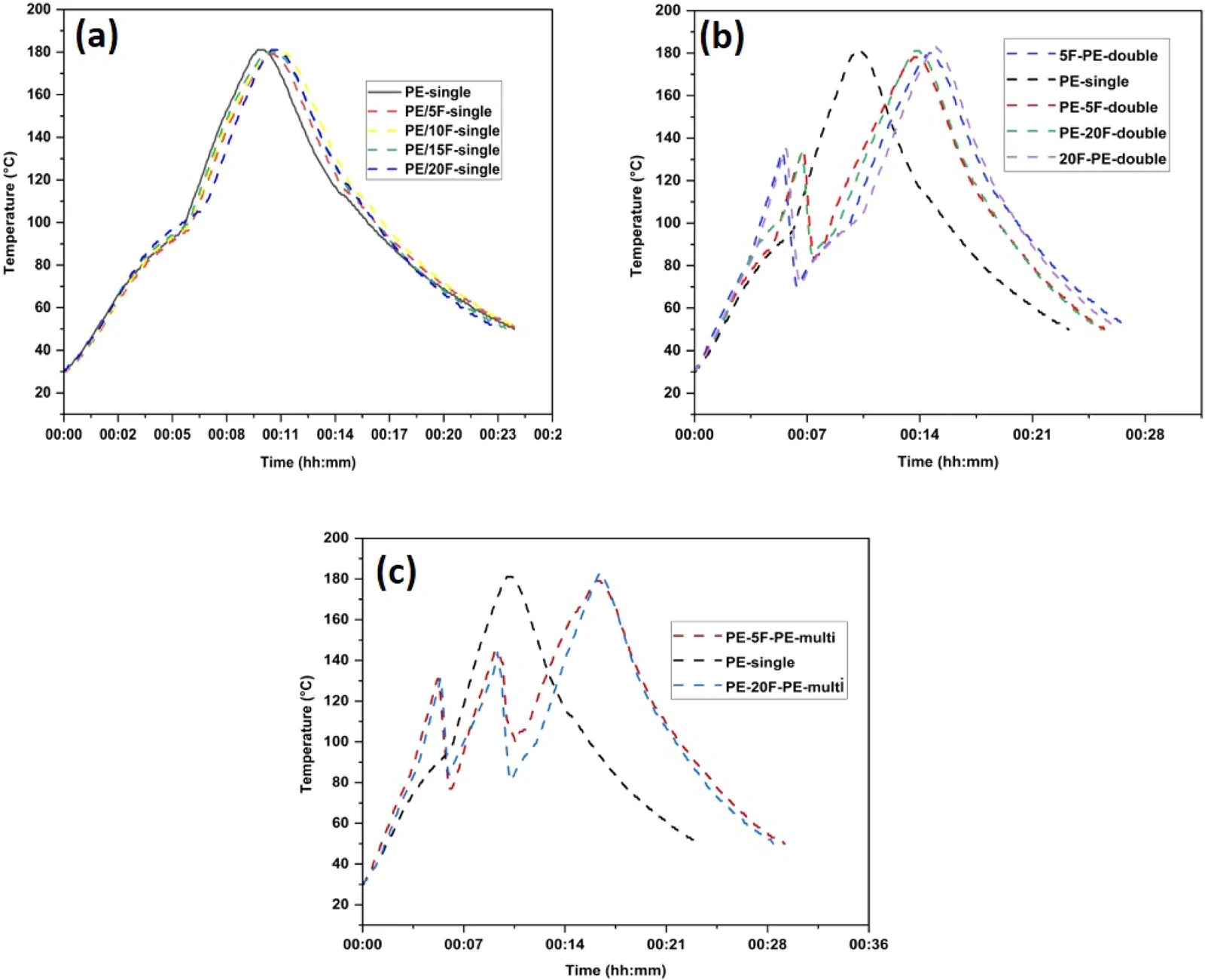
Internal air temperature (IAT) profiles of rotationally molded DPF/LLDPE composites: (a) single-, (b) double-, and (c) multiple (triple)-layered composite configurations.

Within the two double-layered configurations, configurations with an only polymer inside layer (5F-PE-double and 20F-PE-double) required a slightly longer heating time compared to structures with a fiber/polymer inside layer (PE-5F-double and PE-20F-double). This could be due to the quicker heating and sintering of the inside fiber/polymer layer compared to the only polymer layer in the produced composites.

### 3.2 Rheological Analysis

Rheological testing provides insight into the fiber distribution and viscosity of DPF/LLDPE composites for rotational molding, which is a low-shear, external-pressure-free process. Therefore, polymer behavior at low shear rates is critical for the rotational molding process. As shown in Figure 6a, the complex viscosity increased with DPF content, especially at low angular frequencies (e.g., 0.1 rad/s). The viscosity depended on angular frequency, as the curves showed different flow regimes at lower and higher angular frequencies. At low or near-zero shear rates, lower viscosity is preferable for rotational molding for better moldability and sintering process. Incorporation of fibers into the LLDPE matrix, even in powder form, increased fiber–fiber interactions and, together with the polymer, created a network, hence increasing the viscosity compared with LLDPE alone. This effect was also noticed in the storage modulus curves (Figure 6b), wherein storage modulus (G’) values increased with fiber content, indicating greater melt elasticity and stronger fiber–polymer interactions.^2,47^ Loss modulus (G“, Figure 6c) remained higher than storage modulus across frequencies, confirming the primarily viscous nature of the polymer or polymer/fiber melts. However, higher fiber contents led to earlier G’ = G” crossover points and decreasing tan­(δ) (Figure 6d) values with suggesting a transition from viscous to elastic behavior at lower frequencies, indicative of overall good fiber dispersion and composite homogeneity. Overall, the incorporation of 20 wt % DPF resulted in well-distributed fibers within the LLDPE matrix, supporting its suitability for rotational molding of fiber-incorporated composites.^2^

**Figure 6:**
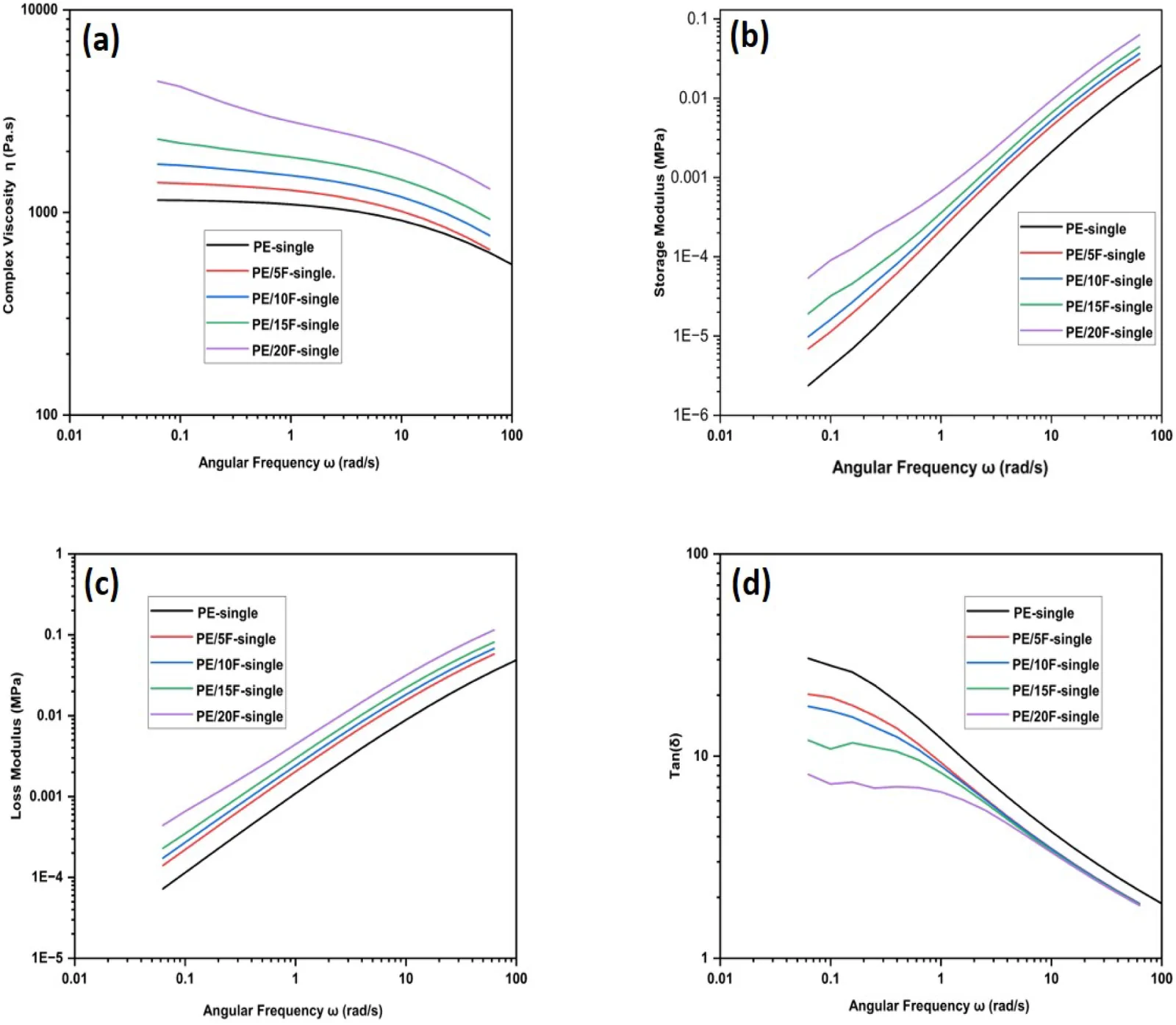
Rheological analysis of DPF/LLDPE roto-molded composites at 190 °C in frequency sweep mode: (a) complex viscosity, (b) storage modulus, (c) loss modulus, and (d) tan­(δ) versus angular frequency curves.

### 3.3 Rotationally Molded DPF/LLDPE Composites and Their Morphology

Rotationally molded LLDPE and DPF-filled LLDPE composites were successfully manufactured, with surface images shown in Figures 7 and 8. In single-layered composites (Figure 7), adding DPF caused the originally whitish LLDPE to appear increasingly yellowish-black. The outer surfaces remained shiny and smooth, with no significant bubbles. Inner surfaces showed rough textures with well-adhered fibers, even at 20 wt % fiber content, no loose fibers were observed. Cross sections confirmed good fiber distribution and fiber/polymer interfaces without significant bubbles. In double-layered structures (Figure 8), samples with outer PE layers (PE-5F-double and PE-20F-double) showed smooth surfaces, while the inner DPF/polymer layers were rough. Conversely, samples with PE as the inner layer (5F-PE-double, 20F-PE-double) displayed smooth inner surfaces, indicating good fiber coverage. Cross sections revealed distinct polymer and fiber/polymer layers with strong bonding and no delamination or voids. For triple-layered structures, the 5 wt % fiber/polymer middle layer (PE-5F-PE) was well sandwiched between PE layers, forming strong interfaces and good adhesion. However, the 20 wt % fiber sample (PE-20F-PE) was poorly covered with polymer in the middle layer due to the insufficient amount of polymer, resulting in visible bubbles, weak interfaces with the outer and inner PE layers, and an overall less compact structure. Thus, adequate polymer content is essential for a high-fiber-content middle layer in triple-layered structures to ensure proper fiber encapsulation and strong interlayer adhesion.

**Figure 7:**
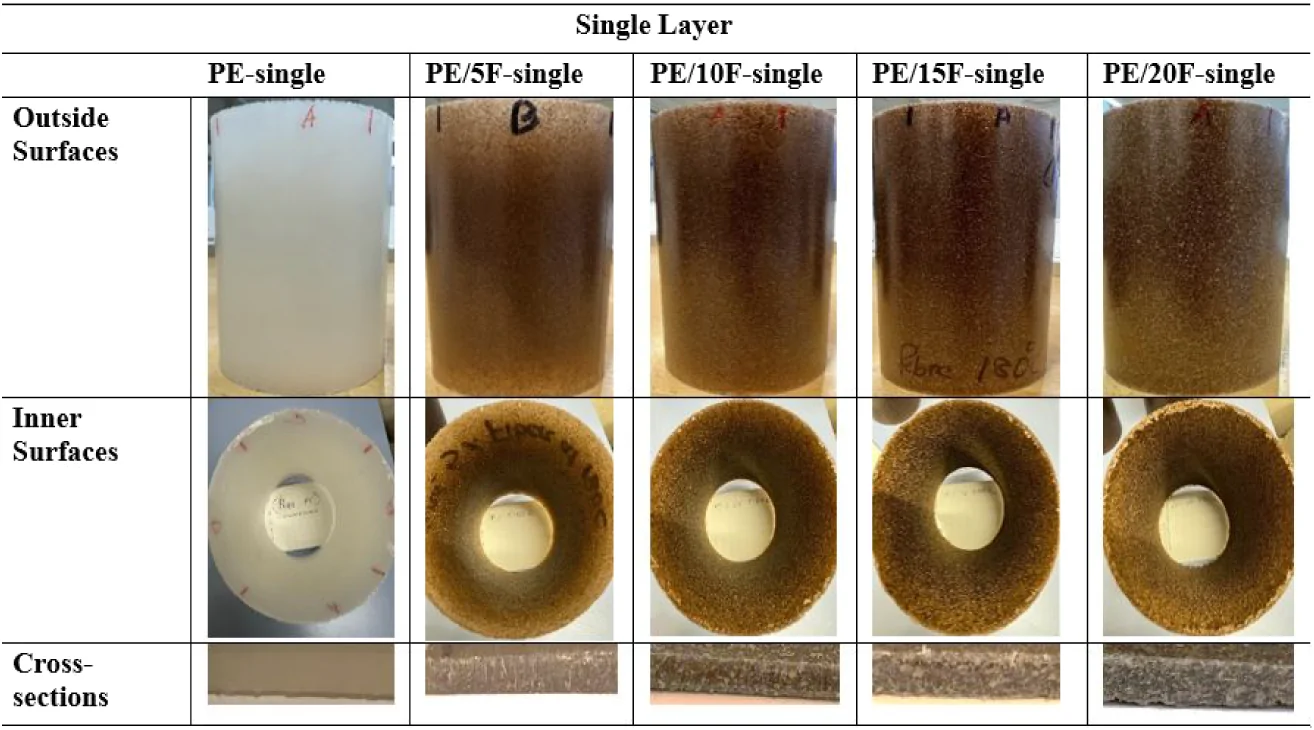
Date palm fiber/LLDPE roto-molded single-layered composites.

**Figure 8:**
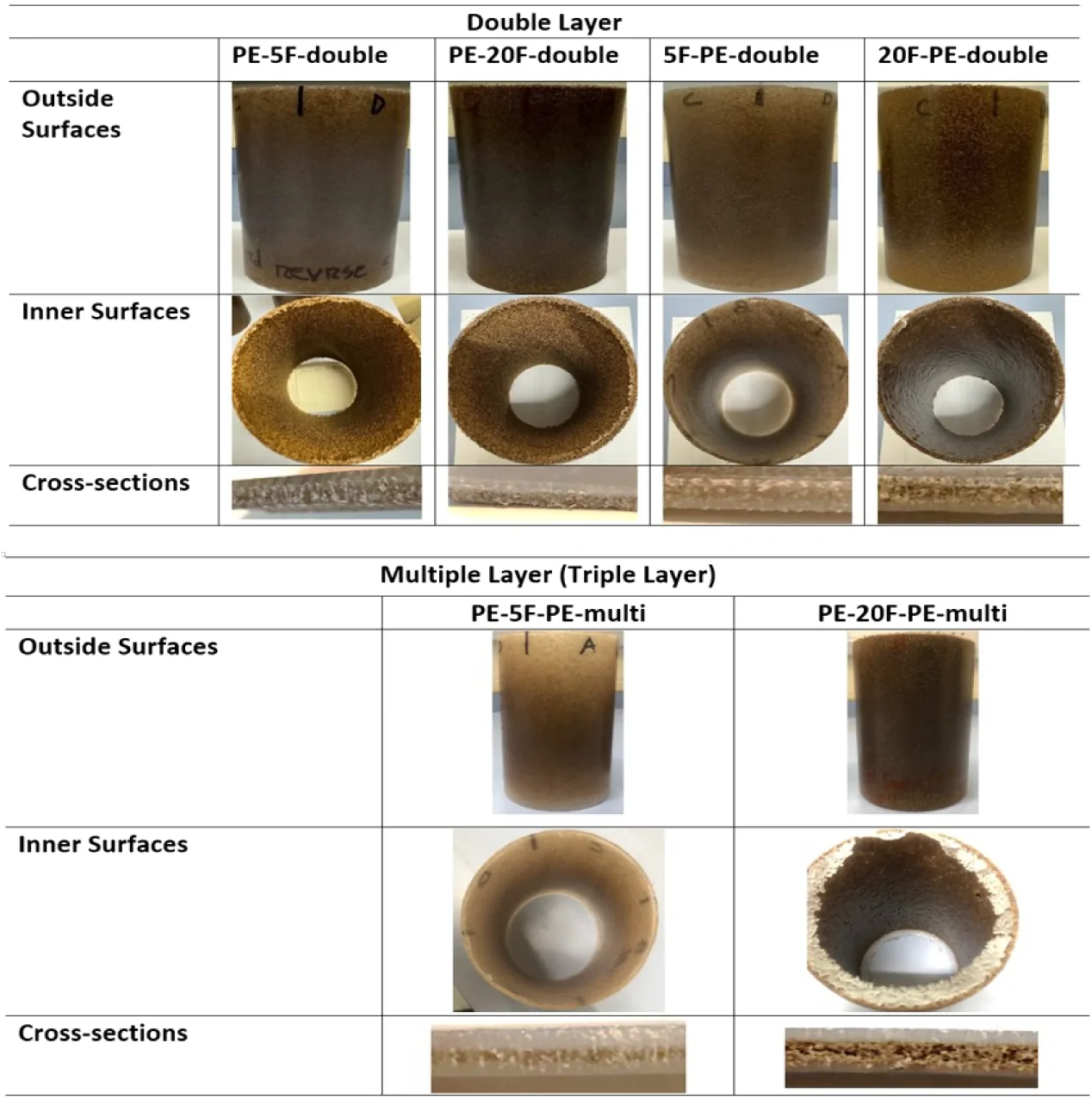
Roto-molded double- and multilayered (triple-layered) date palm fiber/LLDPE composites.

Figure 9 depicts SEM images showing that date palm fibers are cylindrical, with rough surfaces and numerous pores, likely due to incomplete fiber growth or external impurities.^48,49^ Cryo-fractured SEM images of pure LLDPE and DPF-incorporated LLDPE composite samples are also shown in Figure 9. Pure LLDPE displayed a solid, void-free structure. In single-layered DPF/LLDPE samples, fiber dispersion was found to be good and also improved with increasing fiber content. Two types of holes were observed: red-marked holes indicated fiber pull-out from weak fiber–matrix bonding in the cryo-fracturing event, while yellow-marked holes represented bubbles or voids from trapped air attributable to incomplete sintering or gas release during processing. Fewer yellow-marked voids appeared in samples with higher fiber content (20 wt %), supporting the use of more fiber in roto-molded fiber/polymer composites. Similar results were seen in double- and triple-layered samples. In addition, in triple-layered 20 wt % fiber samples, the fibers were not fully covered with polymer in the middle layer due to insufficient amount of polymer to cover the higher fiber content.

**Figure 9:**
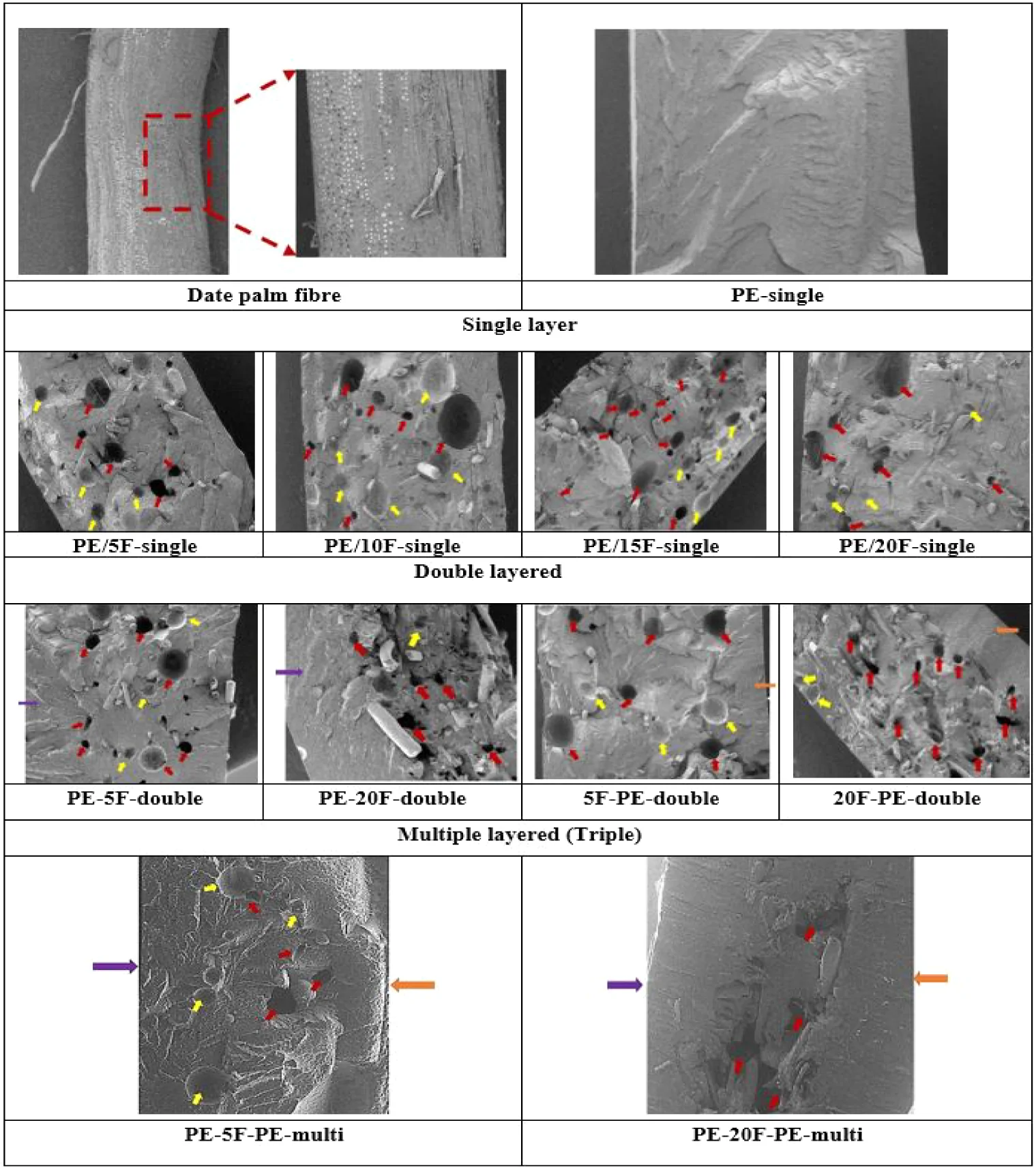
SEM morphological images of date palm fiber and cryo-fractured DPF-filled roto-molded LLDPE composites. All images were taken at a 1 mm magnification scale. (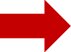) indicates fiber pull-out, (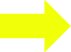) indicates voids or bubbles, (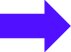) shows the PE layer outside surface, while (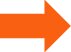) shows the PE layer inside surface of double-layered biocomposites.

### 3.4 XRD Analysis

XRD curves (Figure 10) were identified only for LLDPE and single-layer DPF/LLDPE composites. All of the curves exhibited the characteristic peaks of the orthorhombic crystal structure of PE at around the 2θ positions of 21° (110) and 23° (200).^8,50^ Another small peak was also seen at around 36° 2θ (020), supporting the orthorhombic crystal structure. Nevertheless, a decrease in peak intensity indicated reduced crystallinity and no clear trend in peak intensity changes was found among fiber-added composites. In a previous study, glass fiber was incorporated into a random copolymer polypropylene matrix, where no significant changes in the crystal structure were observed.^51^ However, variations in crystal peak intensity were reported, indicating a reduction in crystallinity. Furthermore, the addition of glass fiber with a particle size of 0.3 mm (300 μm) showed no major effect on crystallite size. In another study, large pineapple fibers were incorporated into an LDPE polymer matrix, where similar observations were made, including no significant alteration in crystal structure alongside reduced crystallinity values.^52^ The analysis of crystallinity changes is presented in the following section.

**Figure 10:**
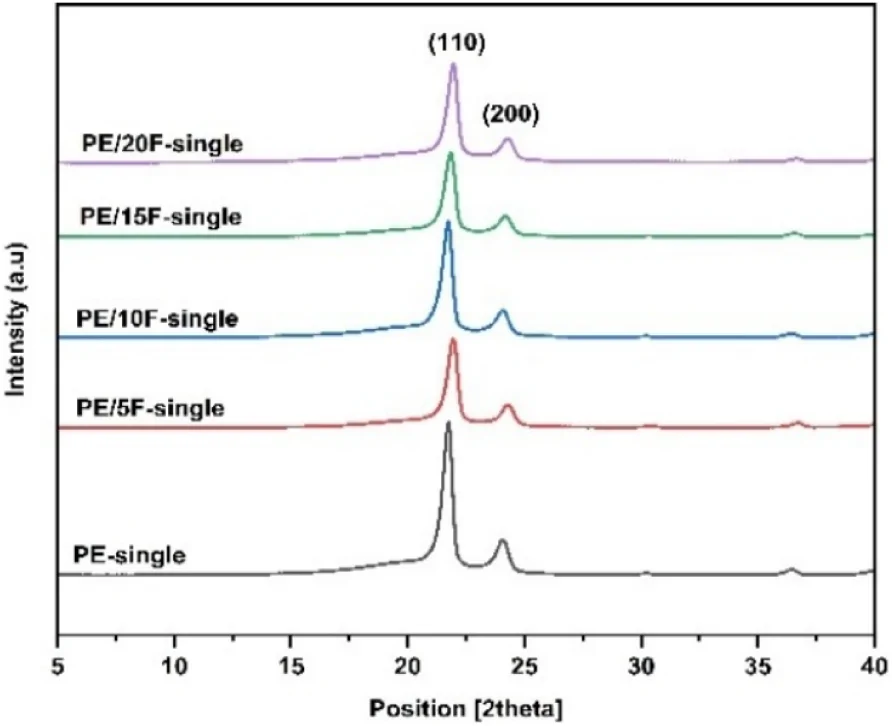
XRD analysis of single-layered rotationally molded DPF/LLDPE composites.

### 3.5 DSC Analysis

DSC analysis (Figure 11, Table 3) showed no significant changes in melting temperatures of DPF-incorporated single-, double-, or multilayered rotationally molded LLDPE composites, even at 20 wt % fiber content. This aligns with the X-ray diffraction results, which showed only the orthorhombic PE crystal structure in all samples. Crystallinity decreased with increasing DPF content in LLDPE single-layer composites, consistent with literature on DPF use in PE,^53^ recycled PP,^54^ and PCL.^15^ For rotational molding, reed-fiber-added PE^55^ and banana-fiber-incorporated metallocene LDPE (mLDPE)^2^ ^2^ also demonstrated lower crystallinity compared to their original polymers. DPF disrupts molecular arrangement, limiting crystallization and crystalline domain formation. DSC cooling-curve analysis (Table 3) revealed that crystallization onset temperatures increased with DPF, while crystallization half-times (time to proceed from the crystallization onset temperature to the peak crystallization point) also lengthened. This suggests that DPF acts as a nucleating agent, initiating crystallization earlier but slowing its overall rate. This could be relevant to the observed decreasing crystallinity values with increasing fiber content in the polymer matrix. A similar trend of crystallinity reduction and longer crystallization half-times with increasing DPF wt % was also seen in double- and triple-layered composites.

**Figure 11:**
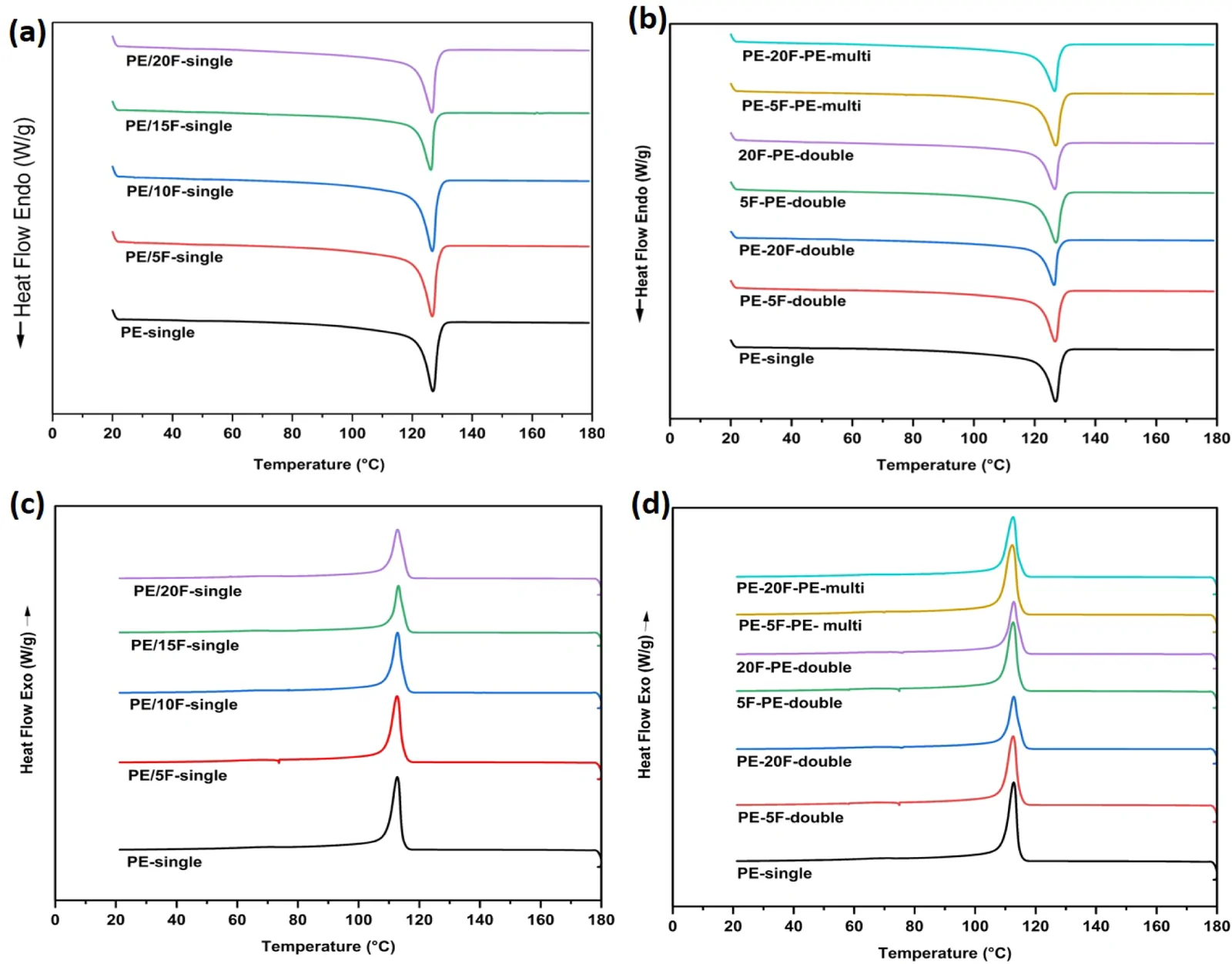
Nonisothermal DSC analysis of DPF/LLDPE composites: heating curves of (a) single-layered and (b) double- and multilayered samples; cooling curves of (c) single-layered and (d) double- and multilayered samples.

**Table 3:** Non-Isothermal DSC Test Data for Melting and Cooling Cycles of DPF/LLDPE Composites

Samples	Melting Temperature (T_m_, °C)	Crystallinity (%)	Crystallization On-Set Temperature (T_0_, °C)	Crystallization Peak Temperature (T_c_, °C)	Crystallization half time (s)
PE-single	126.90	40.00	117.40	112.73	28.02
PE/5F-single	126.70	41.12	117.95	112.45	33.00
PE/10F-single	126.62	39.86	118.36	112.59	34.62
PE/15F-single	126.37	33.50	118.65	112.73	35.52
PE/20F-single	126.56	33.78	118.91	112.60	37.86
PE-5F-double	126.85	39.80	118.10	112.45	33.84
PE-20F-double	126.51	33.10	118.64	112.31	38.00
5F-PE-double	127.10	40.51	118.50	112.18	37.92
20F-PE-double	126.73	35.50	118.86	112.30	39.36
PE-5F-PE-multi	127.00	39.93	117.81	112.18	33.78
PE-20F-PE-multi	126.70	35.66	118.36	112.31	36.30

### 3.6 TGA Analysis

The thermal stability and degradation behavior of DPF/LLDPE samples were evaluated using TGA analysis (Figure 12). Only LLDPE showed a single major degradation step between 400 °C and 500 °C, corresponding to carbon–carbon bond breakdown in LLDPE chains.^53^ The incorporation of DPF into the LLDPE matrix reduced the thermal stability compared with LLDPE, with the greatest reduction observed at 20 wt % fiber content. Generally, date palm fiber shows a three-step thermal degradation process: moisture loss (∼100 °C), pectin and initial cellulose/hemicellulose breakdown (250–350 °C), and major cellulose/hemicellulose degradation (350–500 °C).^15,53,56^ Since the degradation of date palm fiber starts earlier than the LLDPE polymer matrix, the DPF/LLDPE composites showed earlier degradation and reduced thermal stability. This effect was seen for all types of DPF/LLDPE composites.

**Figure 12:**
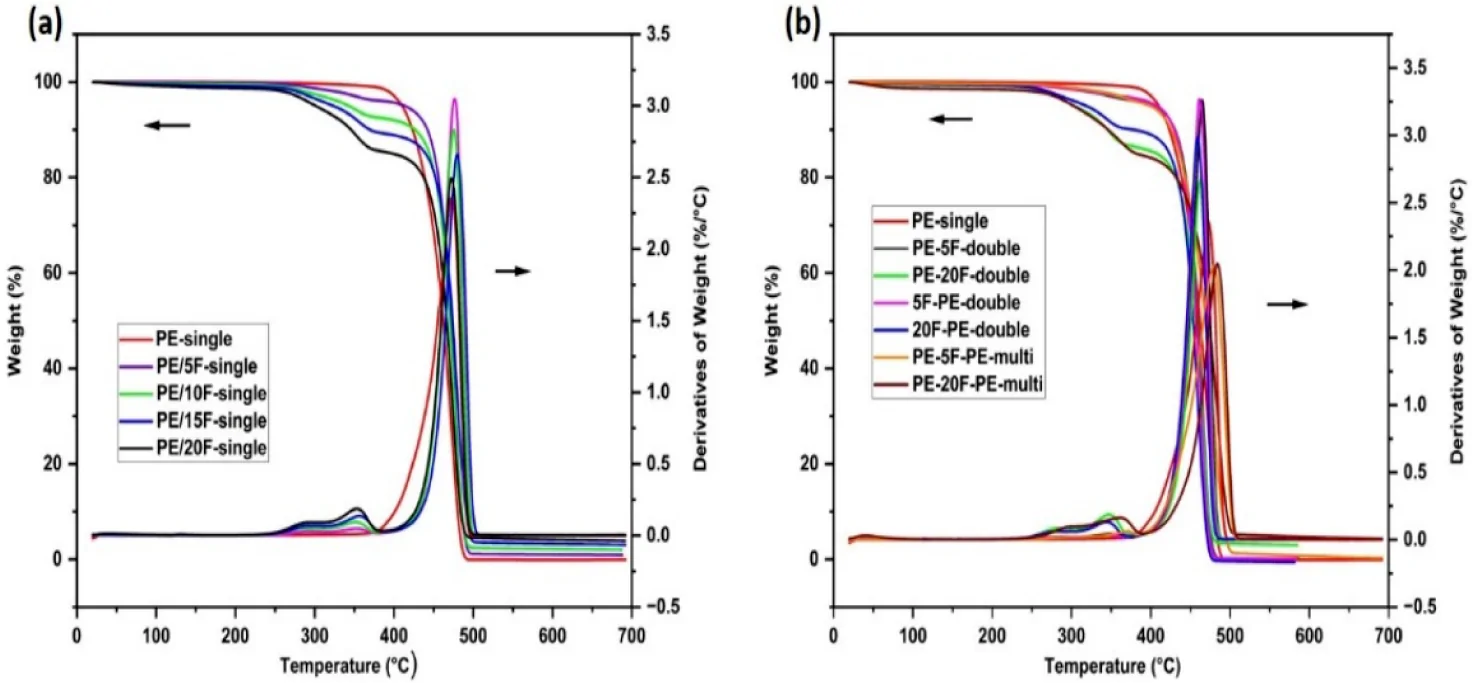
TGA and DTG curves of DPF/LLDPE: (a) single-layered, (b) double- and multilayered composite samples.

### 3.7 Density of Rotationally Molded DPF/LLDPE Composites

The density of the LLDPE-only (PE-single) sample was within the expected range (Figure 13). In single-layered construction, the density values were seen to decrease with up to 10% fiber addition due to the lower density compared to the polymer and the formation of voids/bubbles in the main polymer matrix, as seen in the SEM morphological analysis images. With the increase of fiber contents (15 and 20 wt %), the density values remained stable, likely due to improved sintering that reduced voids, also supported by the SEM image analysis in Section 3.3. Double-layered samples showed higher density than single-layered ones, attributed to longer molding cycles that promote void removal and better compaction. Among double-layered configurations, samples with the PE inner layer had slightly higher density, corresponding to longer heating times. Triple-layered composites with 5 wt % fiber showed the highest density, again due to extended molding cycles. However, at 20 wt % fiber, density dropped sharply due to insufficient polymer in the middle layer, causing fiber/polymer debonding and weaker structures, as confirmed by SEM morphology analysis in Section 3.3

**Figure 13:**
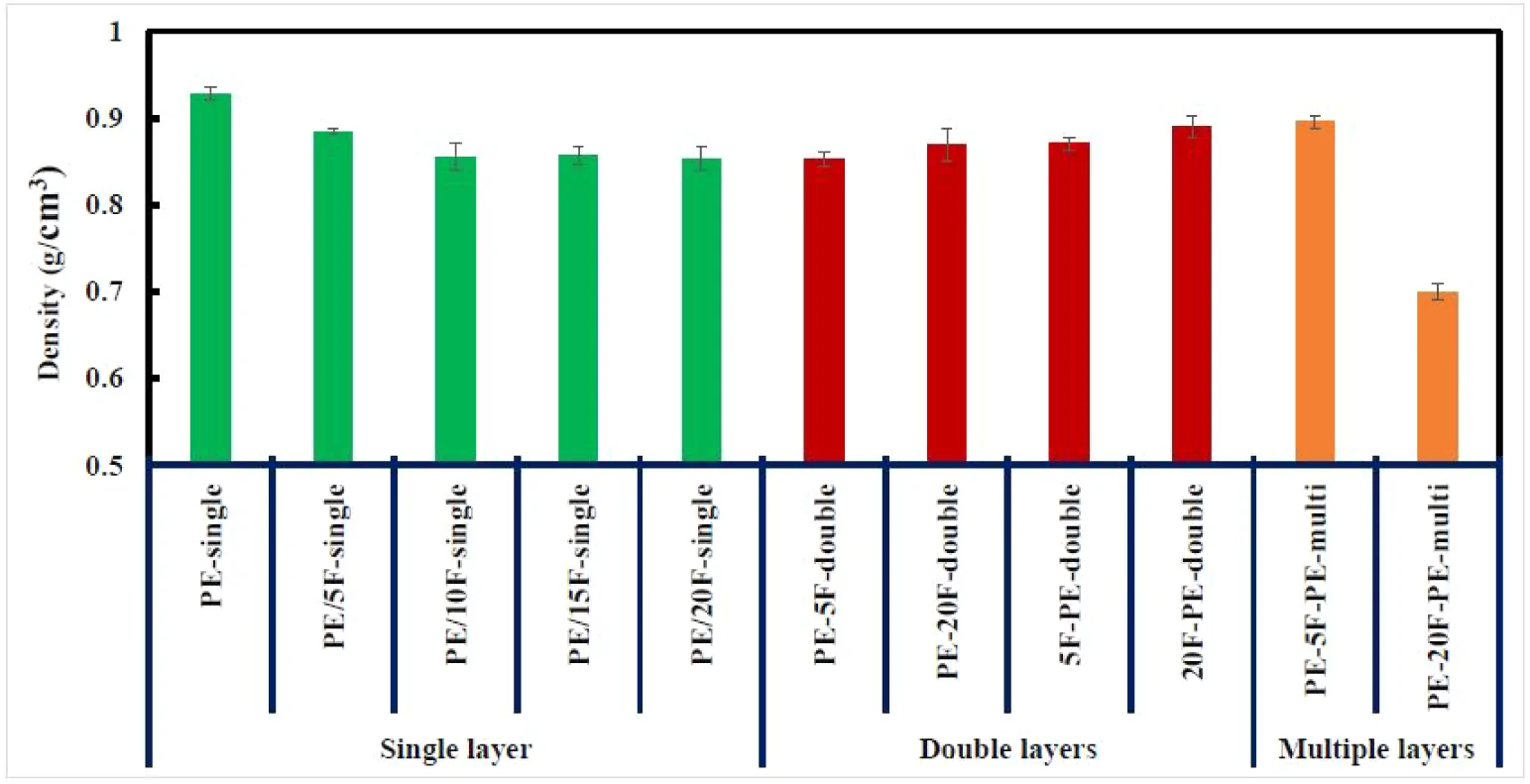
Density of rotationally molded DPF/LLDPE composites.

### 3.8 Mechanical Properties Analysis

In single-layered samples, adding date palm fiber increased the tensile modulus but reduced the tensile strength compared to pure PE samples (Table 4; Figure 14a,b). The PE/20F-single sample showed a 35% increase in modulus (485 MPa vs 360 MPa for PE alone), but a 46% drop in tensile strength. Tensile strain (%) values dropped sharply from 586% (PE) to 30.66% and 12.45% for 5 and 20 wt % fiber contents in single-layered DPF/LLDPE composites, respectively. Fiber addition improved the initial load-bearing capacity but reduced ductility due to weak fiber–matrix bonding, leading to a higher modulus but significantly lower tensile strength and strain values.^13,15^ Such results are expected when hydrophilic fibers are not surface-treated or chemically modified and are mixed with hydrophobic polymers.^9,57^ Similar increases in tensile modulus and reductions in tensile strength were also observed in reed fiber/PE roto-molded composites,^55^ which is because the higher rigidity of the added fibers leads to increased modulus,^7^ whereas a weak fiber/polymer interface reduces the tensile strength. In the literature, the mechanical properties of date palm fibers were found to be good due to their chemical composition (particularly their higher cellulose and lignin contents).^20^ Double-layered samples performed better in tensile modulus and strain values, especially those with a PE inner layer (5F-PE-double, 20F-PE-double), showing an increase in strain (%) compared to their single-layered counterparts. This was due to the specific double-layer configuration and longer molding cycle, which created denser and more compact DPF/LLDPE plastics. The triple-layered 5 wt % fiber sample (PE-5F-PE) had a modulus closer to that of PE alone but achieved a similar tensile strength (17.00 MPa). For tensile strain (%) values, this triple-layered sample also outperformed the single- and double-layered samples by 100% and 28–100%, respectively. This improvement is likely due to the fiber/polymer layer being sandwiched between two PE layers, allowing the structure to behave more like pure PE and resulting in enhanced tensile strength and strain values. However, the 20 wt % fiber content in the PE-20F-PE sample had the lowest modulus and strength, though strain (%) remained the highest (60.50%). This decrease in tensile modulus and strength was attributed to the lower polymer content in the fiber/polymer middle layer, which was insufficient to ensure strong fiber–matrix and layer-to-layer bonding. This effect was also seen in the lowest density values for these 20 wt % fiber-incorporated triple-layered composites. Overall, the addition of date palm fiber (DPF) into LLDPE roto-molded samples did not enhance flexural properties (Table 4). During a flexural test, a combination of compression and tensile forces acts on the tested samples.^58^ Single-layered DPF/LLDPE composites with higher fiber contents exhibited similar or only slightly improved flexural modulus (770 MPa) compared to pure PE samples (760 MPa). In other work, for natural fiber-incorporated PE roto-molded composites, flexural properties did not change significantly for the single layer configuration.^7,55^ However, flexural strength decreased by up to 22% in these composites relative to pure PE. Double-layered samples showed reduced flexural performance compared to single-layered ones, and a similar trend was observed in triple-layered structures. Specifically, triple-layered samples with 5 and 20 wt % fiber content exhibited 18% and 27% lower flexural modulus, respectively, compared to pure PE.

**Table 4:** Tensile and Flexural Properties of Tested Rotationally Molded Date Palm Fiber/LLDPE Composites

Samples	Tensile Modulus (MPa)	Tensile Strength (MPa)	Tensile Strain (%)	Flexural Modulus (MPa)	Maximum Flexural Strength (MPa)
PE-single	360 ± 29	17.50 ± 0.50	500.00 ± 35	760 ± 74	21.26 ± 1.66
PE/5F-single	397 ± 37	13.83 ± 0.28	30.66 ± 2.51	690 ± 44	19.00 ± 0.61
PE/10F-single	436 ± 15	11.80 ± 0.34	18.50 ± 1.56	720 ± 10	18.31 ± 0.26
PE/15F-single	443 ± 33	10.40 ± 0.22	16.10 ± 1.60	770 ± 17	17.51 ± 0.70
PE/20F-single	485 ± 08	09.35 ± 0.18	12.45 ± 1.09	770 ± 43	16.43 ± 0.61
PE-5F-double	504 ± 21	13.28 ± 0.40	31.00 ± 3.31	610 ± 32	16.78 ± 1.73
PE-20F-double	538 ± 36	10.61 ± 1.04	24.18 ± 1.65	705 ± 45	14.61 ± 2.04
5F-PE-double	480 ± 22	14.00 ± 0.37	47.00 ± 4.47	700 ± 46	17.12 ± 0.91
20F-PE-double	470 ± 29	10.32 ± 0.46	38.35 ± 3.00	599 ± 43	14.80 ± 1.81
PE-5F-PE-multi	383 ± 33	17.00 ± 1.80	60.00 ± 3.83	628 ± 47	17.02 ± 0.79
PE-20F-PE-multi	210 ± 24	09.40± 1.09	60.50 ± 5.40	554 ± 15	13.00 ± 1.02

**Figure 14:**
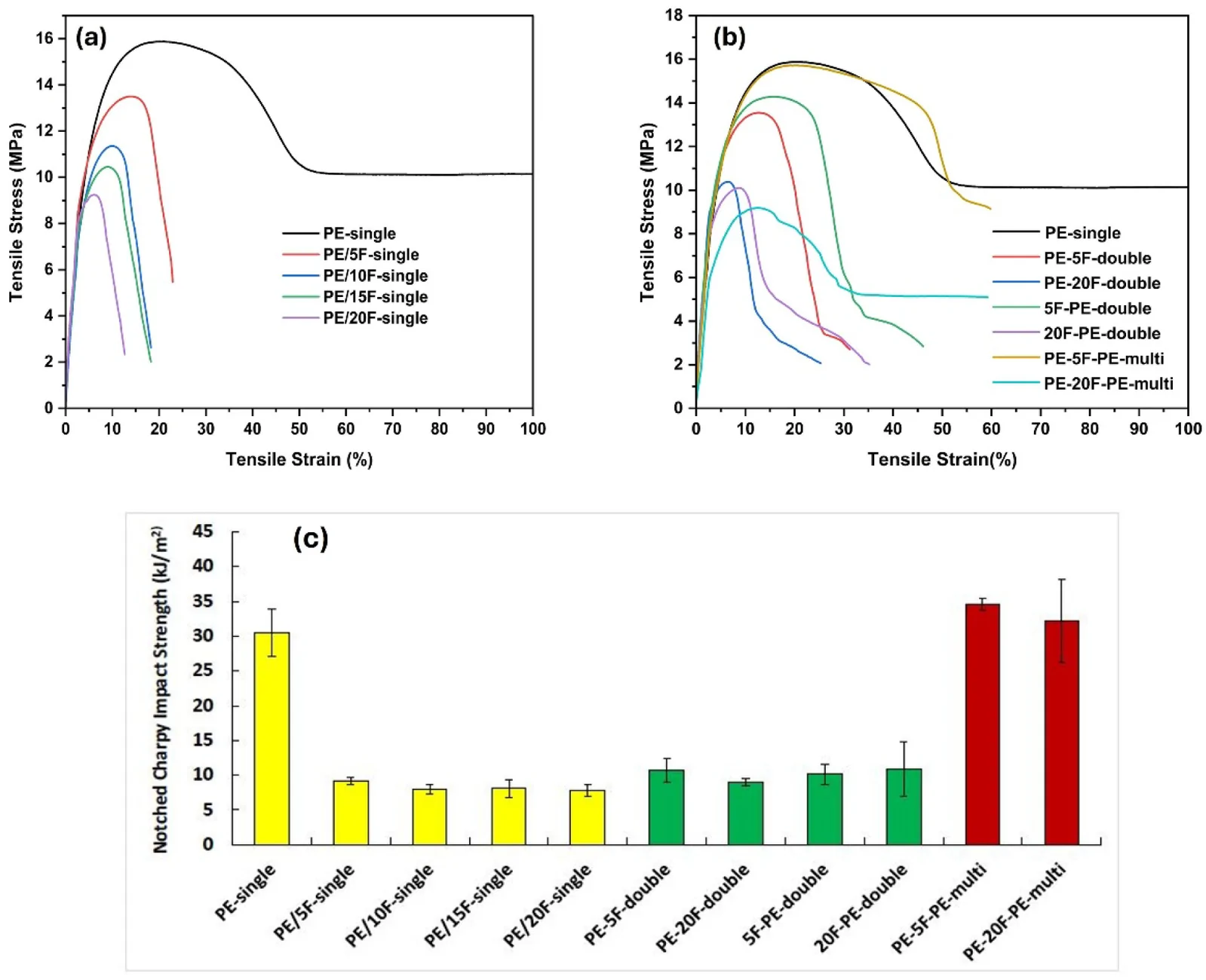
Mechanical properties of DPF/LLDPE—tensile stress–strain curves of (a) single-layered, (b) double- and multilayered composite samples, and (c) notched Charpy impact strength of DPF/LLDPE composite samples.

In Figure 14c, the impact strength of the tested samples was greatly reduced for both single- and double-layered samples. Single-layered samples showed an impact strength of approximately 10 kJ/m^2^ regardless of fiber content, whereas PE-only samples had a much higher value of 30 kJ/m^2^. This lower value, obtained in a dynamic loading scenario (impact test), confirmed the weak bonding at the fiber/matrix interface, which was also seen in previous studies.^2,57^ Double-layered samples exhibited a slight improvement compared with the single-layer samples, but their impact strength remained significantly lower than that of PE-only samples . Interestingly, both 5 wt % and 20 wt % fiber contents in triple-layered structures demonstrated the highest impact strengths of 35 kJ/m^2^ and 33 kJ/m^2^, respectively. These results are also supported by the higher tensile strain (%) values of triple-layered samples. As noted earlier, triple-layered structures behave more like PE-only samples, as the fiber/polymer layer is sandwiched between two PE skin layers and hence the impact resistance increased.

As discussed earlier, fiber addition reduced the crystallinity values, and this trend was observed for all layered composites. The reduced crystallinity values of single-layer samples with high fiber content only supported the lower tensile and flexural strength observed, whereas the lower strain (%) and higher modulus, particularly in tensile properties, were more closely associated with the weak fiber–matrix interface and the rigidity of the added fibers, respectively. For a similar reason, poor impact properties were also observed for single- and double-layered samples. The increase in impact values for three-layered samples and the differences observed in tensile and flexural properties between single-, double-, and triple-layered composites are believed to be related more to their layered configurations than to the crystallinity values reported in Section 3.5 – DSC analysis.

Different failure modes were revealed in the SEM images of tensile-fractured surfaces of DPF/LLDPE composite samples, as shown in Figure 15. All tested samples exhibited these failure modes to varying extents. PE fibrillation under tensile loading was common in all single- and double-layered samples. However, this fibrillation was seen to decrease with increasing fiber content, as the fibers began to share the tensile load alongside the polymer matrix, resulting in higher tensile modulus values, while tensile strain values were significantly reduced. In addition to reduced polymer fibrillation, brittle fiber breakage was also observed, particularly in samples with higher fiber content, contributing to their lower tensile strength in both single- and double-layered configurations. Some bubbles were found, mainly in low-fiber-content samples, and fiber pull-out was observed in all fractured specimens, indicating a weak fiber/matrix interface. Triple-layered samples exhibited the same failure modes under tensile loading conditions.

**Figure 15:**
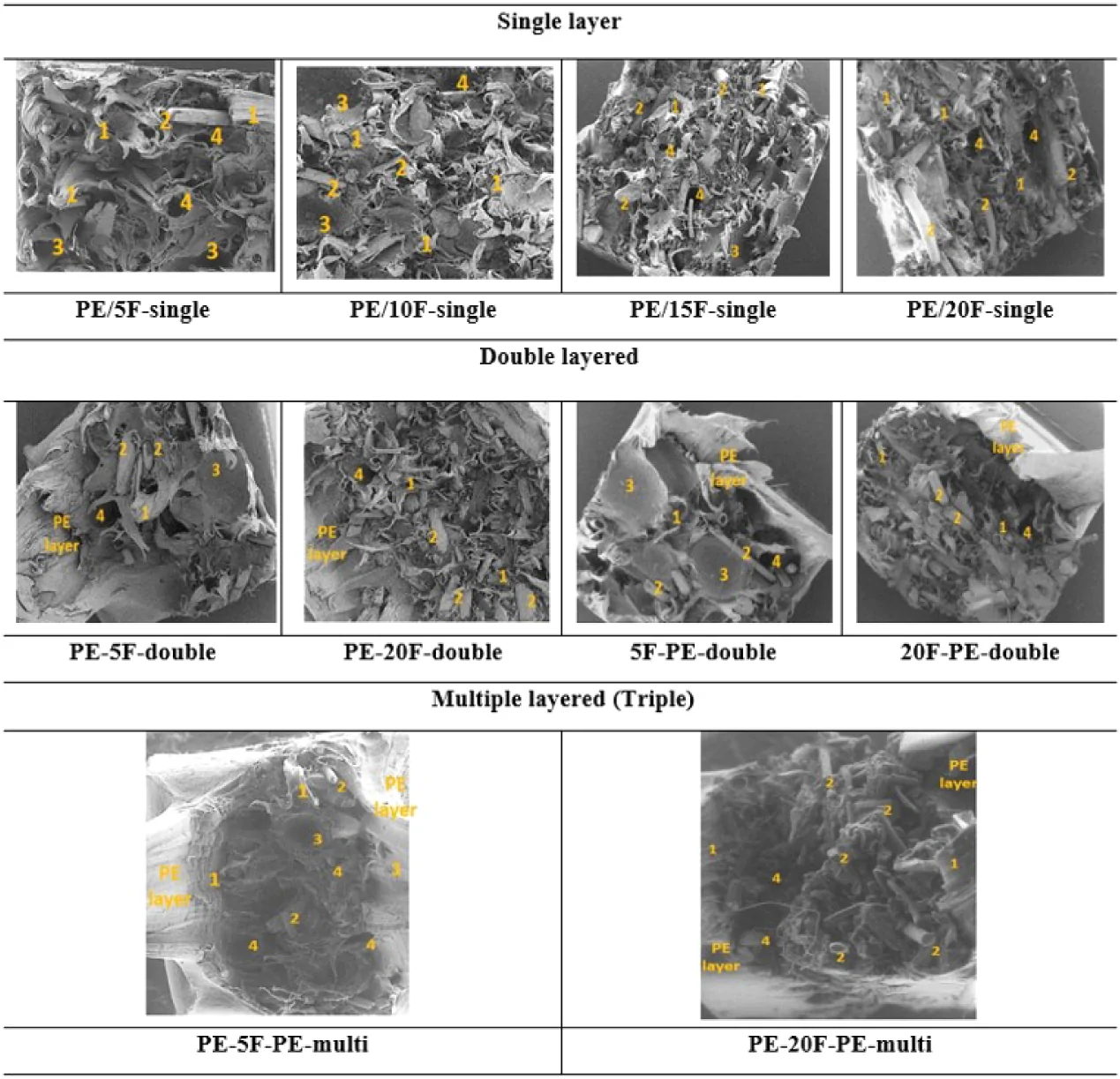
SEM images of the tensile-test fractured surfaces of date palm fiber/LLDPE composites. Different failure modes are numbered as 1= PE fibrillation, 2 = fiber breakage, 3 = bubbles, 4 = fiber pull-out from the matrix.

In the literature, agricultural residue banana fiber was melt-blended with mLDPE (metallocene low-density polyethylene) at 20 wt % into the polymer matrix for single-layered roto-molded samples, while 5 wt % fiber content was used for double- and triple-layered configurations.^2^ A 23% reduction in tensile modulus and approximately 67% reduction in tensile strength were observed in the single-layered sample compared to pure mLDPE. Double-layered samples exhibited similar tensile modulus values to the single-layered samples, while the triple-layered configuration showed a slight improvement, approaching the performance of the pure mLDPE sample. Triple-layered samples demonstrated the highest impact strength at 33 J, although this was still at least 43% lower than that of the pure mLDPE. For flexural properties, the triple-layered samples showed improvements over the single- and double-layered ones, although values remained comparable to the pure mLDPE. In another study, banana fiber was dry-blended with mLDPE at 5 wt % for double- and triple-layered structures, excluding single-layered fiber/polymer samples.^37^ These configurations showed a higher tensile modulus but lower tensile and impact strengths compared to virgin mLDPE. However, flexural modulus values were significantly improved to 430 MPa for double-layered and 577 MPa for triple-layered samples, compared to 236 MPa for virgin mLDPE. Abaca fiber was also investigated in double- and triple-layered PE structures. These samples similarly exhibited increased tensile modulus but reduced tensile and impact strengths compared to pure PE samples.^37^

When comparing the mechanical properties measured in this study to the aforementioned studies in the literature studies, it is evident that DPF/LLDPE samples demonstrated promising performance across single-, double-, and triple-layered structures. Importantly, this investigation explored higher fiber content (up to 20 wt %), whereas previous studies were limited to 5 wt % fiber, specifically for single-layered structures. The results of this study showed that tensile modulus increased with fiber content up to 20 wt % in both single- and double-layered samples. In the triple-layered configuration, even a 5 wt % fiber content maintained a tensile modulus slightly higher than, but closer to, that of pure LLDPE, while achieving similar tensile strength values. Additionally, the 20 wt % fiber triple-layered sample recorded the highest strain among all samples. Most significantly, DPF-valorized triple-layered PE samples (both 5 and 20 wt %) exhibited the highest impact strength, exceeding that of pure LLDPE. This contrasts with previous studies, where agricultural-residue-incorporated samples showed reduced impact strength for single- and multilayered structures. Despite lower flexural properties, particularly in the DPF/LLDPE double- and triple-layered structures with 20 wt % fiber content, the flexural modulus and strength values observed in this study were still comparable to or higher than those reported in previous studies.

### 3.9 Water Contact Angle and Thermal Insulation Analysis

Water contact angle measurements were conducted by placing water droplets on the outer surfaces of single-layered DPF/LLDPE composite samples. The contact angle between the droplet and the composite surface indicates the hydrophilic or hydrophobic nature of the material. This property is particularly important when natural fibers such as date palm fiber (DPF), which are inherently hydrophilic, are used in applications involving high-moisture environments. In Table 5, the neat PE sample showed the highest contact angle (76°), slightly lower than values reported in previous studies, such as 77.8°,^59^ 92°,^60^ possibly due to manufacturing variations. Adding date palm fiber (DPF) reduced the contact angle to 72° and 66° for PE/5F-single and PE/20F-single samples, respectively. This finding was expected since it confirms the increasing hydrophilicity of the composites due to the inherent nature of DPF and the increased surface roughness.^13,15^

**Table 5:** Thermal Conductivity and Water Contact Angle Properties of Single-Layer DPF/LLDPE Composites

Samples	Water Contact Angles (degree)	Thermal Conductivity (W/mK)	Thermal Insulation (m^2^K/W)
PE-single	76.02	0.2740	0.0109
PE/5F-single	71.90	0.2570	0.0116
PE/10F-single	69.12	0.2442	0.0122
PE/15F-single	68.04	0.2344	0.0127
PE/20F-single	66.55	0.2389	0.0125

Thermal conductivity measures the heat conducted through a material under a given temperature difference, and it depends on the components and structure of composites, thickness, and temperature differences between surfaces. If the thermal conductivity of a material increases, the thermal insulation property decreases.^61^ In Table 5, the thermal conductivity of the PE sample was measured as 0.274 W/mK, and this value was found to be very close to the thermal conductivity values of PE reported in the literature, which range from 0.28 to 0.36 W/mK.^62^ The addition of fiber to PE decreased the thermal conductivity values with increasing fiber content in the PE matrix. Hence, 20 wt % fiber in the PE matrix showed the highest insulating property. This was due to the lower thermal conductivity of the date palm fiber, its porous structure, and the voids in the overall composite structure. Incorporation of jute fibers into the PE matrix has also been reported to result in a similar observation. The increase in insulating properties is considered beneficial when date palm fiber–incorporated LLDPE composites are intended for use in applications where insulation is important, for example in building and construction, packaging, and various panels.

From the above results and discussion, it is clear that date palm fiber (DPF) agricultural residue can be successfully valorized to produce rotationally molded LLDPE composites. Both single- and double-layered structures can be developed without any manufacturing issues, with fiber contents of up to 20 wt %. In single-layered composites, date palm fiber contributed to an increased tensile modulus with increasing fiber content while maintaining similar flexural properties. However, impact properties were significantly reduced compared to pure PE samples due to a weak fiber/polymer interface. To overcome this issue, date palm fiber can be treated with alkali, silane, or a maleic anhydride grafting agents, as these treatments have been reported to enhance interfacial properties between natural fibers and hydrophobic polymers.^7,57,63,64^ This approach may also be applied in multilayered rotationally molded composite structures.

Double-layered structures demonstrated improved modulus and strain (%) properties, along with slightly enhanced impact properties compared to single-layered composites. These results support the potential way of using DPF in layered structures for various applications such as water or chemical storage tanks, outdoor furniture, and products sensitive to moisture. In such cases, a fiber/polymer layer should not be exposed directly to real-life environmental conditions. To address this, an additional outer protective PE layer can be added in a double-layered configuration without negatively affecting the mechanical performance of the DPF/LLDPE composites. Water contact angle results indicated an increase in surface hydrophilicity in single-layered composites with increasing fiber content. In this context, the extra PE layer in the double-layered structure is beneficial, helping to mitigate the increased hydrophilicity and maintain performance requirements for moisture-sensitive applications. The incorporation of DPF also enhanced the insulating properties in single-layered composites. It is expected that the double-layered structures will provide even greater insulation due to the added PE layer. Triple-layered structures demonstrated promising results. They are especially useful for rotational molders seeking to utilize DPF agricultural residue in roto-molded composites while opening new opportunities for product development. In terms of tensile properties, triple-layered composites behaved similarly to PE-only samples, while exhibiting the highest impact properties, even outperforming the PE-only samples. This is significant, as it expands the range of potential applications where a combination of good tensile and impact strength is required, such as car bumpers, outdoor furniture, and marine and automotive panels. Additionally, having two polymer layers on the inner and outer skins enhances the composite’s functional performance, particularly in terms of hydrophilic resistance and thermal insulation. However, for triple-layer configurations, it is crucial to ensure sufficient polymer quantity in the middle layer to cover all fibers and form strong interfaces with the two skin layers. In this study, the triple-layer structure with 20 wt % fiber content showed weak interfacial bonding due to insufficient polymer in the fiber/polymer middle layer. This issue can be addressed by either increasing the overall thickness of the composite to allow more polymer coverage in the middle layer or by adjusting the layer configuration, reducing the thickness of the skin layers and increasing the polymer content in the fiber/polymer middle layer, especially for 3-mm-thick samples.

## 4 Conclusions

Date palm fibers (DPF) were valorized for the first time in rotationally molded LLDPE composites, with various fiber contents and layered configurations (single/double/triple layers). The results indicate that agricultural residue DPF can be successfully used in both single- and multilayered composites. This broadens the potential for product development across a range of applications in rotational molding industries.

Double- and triple-layered composites required 10–27% longer molding cycles than single-layer ones due to additional layer processing. No major manufacturing issues occurred in single- or double-layered structures, but triple-layered composites with 20 wt % fiber required additional polymer to fully cover the fibers, leading to weak interfacial bonding and poor fiber–polymer adhesion. Fiber addition reduced composite density compared to neat PE, while multilayered structures showed slightly higher densities due to more compact formation. SEM confirmed uniform fiber distribution in the LLDPE polymer matrix, consistent with rheological results. XRD showed no changes in crystal structure, while DSC indicated slower crystallization and reduced crystallinity with higher fiber content. TGA revealed earlier thermal degradation in DPF/LLDPE composites, especially at higher fiber contents. Single-layered composites exhibited higher tensile modulus but lower strength, strain, and impact properties with fiber addition. Double-layered composites exhibited improved modulus and strain, while triple-layered composites demonstrated tensile behavior similar to neat LLDPE and achieved the highest impact strength. All composites showed good flexural performance compared to similar natural or agricultural biomass fiber-filled roto-molded composites reported in the literature. Fiber addition increased hydrophilicity in single-layered samples, suggesting that double- or triple-layered structures may better protect moisture-sensitive applications. Higher fiber content also enhanced thermal insulation, with additional gains expected in layered configurations.

Valorizing date palm fibers offers a sustainable way to partially replace virgin polymer in roto-molded LLDPE composites, improving their environmental profile. While this study used up to 20 wt % fiber content, future work will investigate higher fiber contents, fiber surface modifications to further enhance properties, and the long-term mechanical properties (fatigue, aging, hygroscopic behavior, etc.) of these composites.
